# Atopic Disease as a Risk Factor for Recurrent Herpetic Keratitis

**DOI:** 10.3390/microorganisms12010220

**Published:** 2024-01-21

**Authors:** Margarita Safir, Michael Mimouni

**Affiliations:** 1 Ophthalmology Department, Yitzhak Shamir Medical Center, Sackler Faculty of Medicine, Tel Aviv University, Tel Aviv 69978, Israel; sapir.margarita@gmail.com; 2 Department of Military Medicine, Faculty of Medicine, The Hebrew University of Jerusalem, Jerusalem 91905, Israel; 3 Ophthalmology Department, Rambam Health Care Campus, Haifa 3109601, Israel

**Keywords:** herpes simplex virus, keratitis, atopic conjunctivitis

## Abstract

Recurrent herpetic keratitis is a leading cause of blindness worldwide. In this population-based cross-sectional study, the medical records of Israeli adolescents and young adults who underwent systematic preconscription evaluation for mandatory military service were reviewed. The prevalence of atopic conjunctival disease was evaluated in cases with and without documented recurrent herpetic keratitis. The association was tested using uni- and multivariant analyses. Overall, 940,892 adolescents and young adults were included. The mean age was 17.57 ± 1.50 years (range 16–20 years), and 40.70% of participants were female. Recurrent herpetic keratitis was documented in 160 cases, with a prevalence of 0.017% in this age group. Compared to the general population, patients with recurrent herpetic keratitis were significantly more likely to be males (*p* = 0.003) with a concomitant diagnosis of atopic conjunctival disease (*p* < 0.0001). Patients with atopic conjunctival disease were 10.60-fold more likely to experience recurrent herpetic keratitis (95% confidence interval (CI): 6.76–16.64, *p* < 0.0001). Upon multivariate analysis, the results remained significant (*p* < 0.001). Cases of severe atopic conjunctival disease were more prone to recurrent HSV keratitis compared to mild cases (*p* < 0.001). These findings suggest that the timely appropriate treatment of atopic conjunctival disease may help reduce the frequency and severity of recurrent HSV keratitis and its complications.

## 1. Introduction

Herpes simplex virus (HSV) infection is a global health concern, affecting over 5 billion people worldwide [1]. Among the numerous manifestations of HSV infection, ocular involvement presents a particularly menacing threat. It is estimated that one in five individuals afflicted with ocular HSV infection will ultimately develop stromal keratitis, a severe and vision-threatening condition [2]. In fact, herpetic keratitis is considered to be the leading cause of monocular infectious blindness due to stromal opacification in developed countries [3], causing 40,000 new cases a year of severe monocular visual impairment or blindness worldwide [4]. Beyond the ominous threat of blindness, HSV keratitis can manifest in various debilitating ways, including dry eye disease, persistent pain, corneal surface irregularities, ulcerations, and, in rare instances, corneal perforation [5]. Thus, this highly prevalent disease not only exacts a profound toll on patient well-being but also imposes a substantial burden on healthcare systems globally [6].

Each additional episode of herpetic keratitis increases the risk of future episodes [2,7], further increasing the likelihood of severe visual impairment [7,8]. Young et al. found that the risk of recurrence was 27%, 50%, and 57% at one, five, and ten years after the primary keratitis event, respectively, and increased to 38% and 67% at one and five years, respectively, if the initial episode was followed by recurrence [8]. Thus, early diagnosis, timely correct treatment, and modification of preventable predisposing factors for recurrence are mandatory. Unfortunately, HSV keratitis may sometimes pose a diagnostic challenge in a patient with no prior history of HSV keratitis, mimicking multiple other common ocular conditions [6]. In such cases, obtaining a thorough medical history and considering known predisposing factors for HSV keratitis is of great value in facilitating a rapid and correct diagnosis.

Currently known predisposing factors for HSV keratitis include immune system compromise [3,7,9], ultraviolet (UV-A) radiation exposure [9], trauma to the ocular surface [10,11,12,13], and various topical medications [3,14]. Atopic dermatitis, a highly prevalent dermatological condition, has been linked to an increased risk of cutaneous [15] as well as ocular [16,17] HSV infection. However, to the best of our knowledge, no large-scale study has evaluated the association between atopic conjunctival involvement and recurrent HSV keratitis. The investigation of such an association is especially important for young patients, where atopic disease is more prevalent and the consequences of recurrent HSV keratitis have the potential to affect patients’ vision and quality of life for many years. Consequently, the primary aim of our study is to investigate whether atopic conjunctival involvement plays a significant role in the recurrence of HSV keratitis, shedding new light on this critical aspect of the disease’s pathogenesis in adolescents and young adults.

## 2. Methods

### 2.1. Ethics

This study was approved by the Institutional Review Board of the IDF medical corps (IRB number 2372-2023, 23 September 2023) and adhered to the tenets of the Declaration of Helsinki. The participants’ anonymity was preserved. Patient consent was waived as the raw data was deidentified.

### 2.2. Patient Evaluation

Military service is mandatory in Israel. Thus, the entire Israeli adolescent population undergoes a standardized preconscription medical evaluation. Israeli military preconscription assessment is based on standardized medical evaluation followed by an appropriate fitness-for-service (FFS) code assignment, taking place between 16 and 18 years of age. This medical evaluation includes questionnaires filled by the applicants and their family physician, along with visual acuity and full slit lamp examination conducted by an ophthalmologist. Based on this data, one or more FFS classification numerical codes are assigned to the candidate according to their medical status. The FFS codes are indicative of both the medical diagnosis (or similar diagnoses grouped by pathogenesis) and disease severity. After completion of the aforementioned evaluation, candidates are deemed either fit or unfit for military service, with all their medical data recorded in the military medical file regardless of their fitness for further military service.

Mandatory military service lasts 2 years. During this period, the medical status of an individual may change, with the diagnosis of new medical conditions or an increase/decline in the severity of a preexisting medical condition. In such a case, a new FFS numerical code is assigned to the patient based on the new medical status. Thus, at a given point, the individual’s FFS codes represent his most updated medical status.

### 2.3. Herpetic Keratitis Diagnosis

An FFS code for recurrent HSV keratitis is assigned in a two-point scale according to episode frequency and the presence of visually significant resultant corneal opacification or scarring. The information required for this FFS code assignment is gathered from a certified ophthalmologist’s examination and documentation in the patient’s medical file in the past. The diagnosis of HSV keratitis was at each ophthalmologist’s discretion, and data regarding the clinical course and laboratory evaluation leading to such diagnosis for each patient was not available since it was performed prior to military service. The FFS code for recurrent HSV keratitis is assigned when at least one eye of the patient is documented to be affected by recurrent HSV keratitis; thus, no data regarding a single episode or bilaterality is available based on FFS code examination.

### 2.4. Atopic Conjunctival Disease Diagnosis and Grading

An FFS code for atopic conjunctival involvement is assigned following an ophthalmic evaluation. Atopic conjunctival involvement may be either vernal conjunctivitis or atopic conjunctivitis. Regardless of the underlying pathology, the disease severity is determined according to corneal involvement and the frequency of exacerbations. For the purpose of FFS code assignment, a mild–moderate disease was defined as mild blepharoconjunctivitis with mild functional disturbance and short infrequent exacerbations that were well-controlled by topical non-immunomodulating treatment (topical steroids during exacerbations allowed). Severe disease was defined as blepharoconjunctivitis with multiple documented exacerbations, evidence of significant ocular involvement, and functional disturbance or the need for immunomodulating topical treatment for disease control (either cyclosporin or tacrolimus).

### 2.5. Data Gathering and Patient Selection

Medical records of all patients who were evaluated for and/or served in the IDF between January 2011 to December 2021 were reviewed. Collected data included demographic characteristics (age at FFS assignment, gender, height, weight, years of education, and cognitive function scores), FFS codes, and their assignment dates. Overall, 3.481 cases were excluded due to incomplete FFS information.

Socio-demographic and anthropometric data were recorded as part of the preconscription intake process. Education was grouped according to the number of years of formal schooling: <9, 10 to 11, and >12 (which includes higher and academic studies). Participants underwent a comprehensive cognitive assessment through a battery of tests for verbal and non-verbal intelligence that yielded a cognitive function score (CFS). This score is normally distributed in the population, ranging from one to nine. CFS is considered a valid measure of general intelligence, and is highly correlated with the Wechsler Adult Intelligence Scale [18]. Body mass index (BMI) was calculated as weight in kilograms divided by height in meters squared. BMI and height values were coded according to the age- and sex-adjusted growth charts of the United States Centers for Disease Control and Prevention (CDC) [19].

Additional conditions which were tested for in the current study as potential risk factors for recurrent herpetic keratitis were diabetes mellitus, autoimmune conditions, human immunodeficiency virus infection, and atopic dermatitis. FFS codes are assigned for these conditions as part of preconscription evaluation and during military service according to disease severity and need for treatment. For autoimmune diseases, separate FFS codes are assigned by groups of diseases: rheumatic joint disease, vasculitis, gout, inflammatory bowel disease, and familial Mediterranean fever. For the purpose of analysis, each FFS was tested separately. For patients with diabetes mellitus, a single FFS code encompasses both type 1 and type 2 diabetes.

### 2.6. Statistical Analysis

Statistical analysis was performed using IBM SPSS Statistics 25 (SPSS Inc., Chicago, IL, USA). For categorical variables, the χ2 test was used. Clinical parameter distributions were tested for normality using the Shapiro–Wilk test. An independent *t*-test was conducted for continuous variables with a normal distribution and the Mann–Whitney U-test was used for variables with a non-normal distribution. Binary logistic regression was used, controlling for universal possible confounders, following the best subset method. *p*-values less than 0.05 on a two-sided test were considered statistically significant. Unless otherwise specified, data are presented as mean ± standard deviation (SD).

The sample size was calculated based on a 149 per 100,000 prevalence of a history of herpetic infection [20] and an up to 57% risk of recurrence [8], adding up to an estimated prevalence of 0.09% of recurrent HSV keratitis in the general population. In order to detect a four-fold-increased odds ratio for recurrent HSV keratitis among the exposed group with 80% power and 5% alpha, 60 cases were needed in each group, with a total of 120 cases.

## 3. Results

The medical files of 940,892 patients were included. The mean age at FFS assignment was 17.57 ± 1.50 years (range 16–45 years, median 17 years, 99.44% age 25 years or younger), and 40.73% of participants were female. Recurrent herpetic keratitis was documented in 160 cases, with a calculated prevalence of 0.02%.

The demographic characteristics of cases with and without recurrent herpetic keratitis are described in Table 1. Patients with recurrent HSV keratitis were more likely to be males compared to the general population (70.63% versus 59.27%, respectively, *p* = 0.003), with an odds ratio (OR) of 1.65 for male gender (95% confidence interval (CI): 1.18–2.32, *p* = 0.004).

Overall, 6122 cases of documented atopic conjunctival disease were found in this cohort, with a prevalence of 0.65%. Univariate analysis revealed an increased tendency for recurrent HSV keratitis in patients with atopic conjunctival disease compared to the general population (0.42% compared to 0.04%, *p* < 0.001), with a 10.60-fold increased risk for HSV keratitis in this group (95% confidence interval (CI): 6.76–16.64, *p* < 0.0001). Upon multivariate analysis, after controlling for age and gender, the results remained statistically significant (*p* < 0.01).

**Figure 1:**
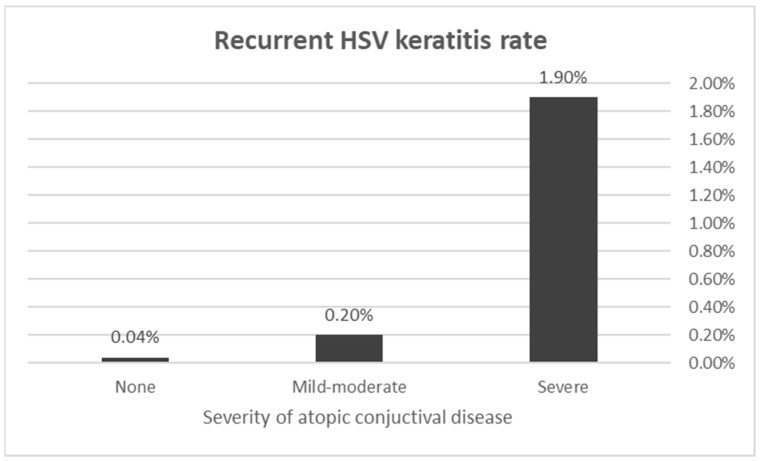
Recurrent herpes simplex virus (HSV) keratitis occurrence according to atopic conjunctival disease severity. Patients with both mild–moderate and severe conjunctival disease were at a significantly increased risk to develop recurrent HSV keratitis compared to the general population (OR 4.30, 95% CI: 2.03–9.09, *p* < 0.0001 and OR 50.41, 95% CI: 28.84–88.10, *p* < 0.0001, respectively).

When comparing the atopic disease severity of patients with and without recurrent HSV keratitis, patients in the HSV keratitis group had worse atopic conjunctivitis grading (when affected) compared to the general population (2.00 ± 0.00 versus 1.14 ± 0.34, *p* < 0.0001) (Figure 1). Stratification according to the severity of atopic conjunctival disease yielded a 4.30-fold risk for recurrent HSV keratitis in the mild–moderate atopic conjunctivitis group compared to the general population (95% CI: 2.03–9.09, *p* < 0.0001) and a 50.41-fold risk in the severe atopic conjunctival disease group (95% CI: 28.84–88.10, *p* < 0.0001).

Additional potential risk factors for recurrent herpetic infection were assessed, including the concurrent diagnosis of autoimmune disease, diabetes mellitus, human deficiency virus infection, and atopic dermatitis, with no significant association found (*p* > 0.05 for all).

## 4. Discussion

Herpes simplex virus (HSV) is a ubiquitous pathogen in the general population, with 20% of children and over 60% of adults reported to be seropositive for the virus [15]. Ocular involvement results mostly from a reactivation of the virus from its dormant state in the trigeminal ganglion, having tracked in a retrograde fashion to the ganglion from the lip along the mandibular branch of the trigeminal nerve [21]. Ocular HSV infection exhibits a spectrum of ocular manifestations that significantly impact visual health, including conjunctivitis, keratitis, uveitis, and retinitis, reflecting the virus’s capacity to affect multiple ocular tissues [13,22]. The intricacies of these ocular manifestations involve dynamic interactions between viral factors, host immune responses, and environmental triggers.

Among the diverse ocular presentations, herpetic keratitis stands out as a primary concern, recognized for its potential to cause stromal opacification and subsequent blindness. This manifestation often results from recurrent viral reactivation within the cornea, leading to a cascade of inflammatory responses and tissue damage [23]. HSV keratitis encompasses a spectrum of clinical manifestations, each presenting unique challenges in diagnosis and management. The three main types of HSV keratitis are epithelial keratitis, stromal keratitis, and endothelial keratitis. Epithelial keratitis primarily affects the superficial layer of the cornea, the epithelium. It is characterized by the presence of dendritic or geographic corneal ulcers, a hallmark of active viral replication. Epithelial keratitis is often recurrent and may manifest with symptoms such as pain, photophobia, and tearing. Prompt diagnosis and treatment, typically with antiviral medications, are crucial to prevent post-infection neuralgia and shorten the course of the disease. In stromal keratitis, the infection infiltrates the corneal stroma, leading to inflammation and potential scarring. This type poses a significant risk for visual impairment and is often associated with immune-mediated responses [23]. Management involves a combination of antiviral therapy and topical steroids to control the stromal inflammatory process. The third type of HSV keratitis, endothelial keratitis, affects the corneal endothelium, the innermost layer of the cornea. Endothelial keratitis may result in corneal edema due to compromised endothelial cell function. Management of endothelial keratitis involves antiviral therapy and frequently applied topical steroids with close ophthalmological follow-up. In cases of significant endothelial compromise, corneal transplantation may be considered following resolution of the acute phase of the disease.

Visual impairment due to recurrent HSV keratitis may occur through several mechanisms [2,7,23]. The most common and concerning is the development of corneal opacification and scarring, particularly in cases of stromal keratitis. This structural damage can significantly compromise the transparency of the cornea, leading to visual disturbances and, in severe cases, profound vision loss. Additionally, the chronic inflammatory milieu associated with recurrent episodes may contribute to the development of immune-mediated complications such as corneal neovascularization and persistent epithelial defects, further exacerbating visual impairment. Moreover, the corneal nerve damage induced by HSV infection may result in neurotrophic keratopathy, characterized by decreased corneal sensitivity and impaired healing, posing an additional challenge in maintaining ocular health and visual acuity. Finally, endothelial decompensation may occur, leading to chronic corneal edema and subsequent opacification.

The differential diagnosis of HSV keratitis is a critical aspect of clinical evaluation, considering its potential overlap with various ocular conditions, including other types of infectious keratitis (caused by bacteria, viruses, fungi, or acanthamoeba), immune-mediated keratitis, and neurotrophic keratitis [22,24]. Since ocular manifestations of recurrent corneal HSV reactivation may endanger a patient’s visual function, it is prudent to recognize and correctly manage these reactivations as soon as possible. In order to accomplish this goal, one should be familiar with factors predisposing patients to recurrent HSV keratitis.

Local or systemic immune system compromise is a well-established contributor to recurrent episodes.

Exposure to ultraviolet (UV-A) radiation, a ubiquitous environmental factor, has been linked to the reactivation of herpes simplex virus in some studies, presumably by means of local immunosuppression [25], underscoring the role of environmental influences in disease recurrence. Trauma to the ocular surface, whether through direct injury or surgery, stands as another recognized risk factor [10,11,12,13,21]. Additionally, certain topical medications have been identified as potential contributors, including immunomodulatory compounds and prostaglandin analogues used for glaucoma management [23]. The reported increased prevalence and severity of herpetic keratitis in individuals with atopic conditions underscores the significance of atopic disease in this context [26]. However, despite the cumulative knowledge, there remains a notable gap regarding the association between atopic conjunctival involvement and recurrent herpes keratitis.

Atopic conjunctival disease is a highly prevalent ocular disorder affecting up to 10.1% of the general population [27,28], with different prevalence according to age. A recent large-scale study reported its prevalence to be 0.3%, 6.6%, 18.3%, 15.8%, 8.1%, and 4.9% in infancy (<1 years), toddlerhood (1–2 years), early childhood (3–5 years), middle childhood (6–11 years), early adolescence (12–18 years), and late adolescence (18–21 years), respectively [28]. The prevalence of atopic conjunctival disease in the current study was on the lower side of previous reports (0.65% versus 0.003–7.3%) [27,28], probably due to some amount of reporting bias, where only patients who were symptomatic during a short time preceding pre-conscription evaluation were assigned the corresponding FFS code.

The current study demonstrated a significant association between atopic conjunctival disease and recurrent HSV keratitis in a large cohort of immunocompetent adolescents and young adults. The exact mechanism underlying the association between atopic conjunctival disease and recurrent HSV keratitis remains unclear. However, it is plausible that the chronic inflammation and immune dysregulation characterizing atopic conjunctival disease could predispose patients to more frequent herpetic keratitis episodes [29,30]. For instance, it has been shown that the altered immune response in atopic individuals may facilitate viral reactivation and replication, as well as impair the host’s ability to control HSV infection [15,31]. This hypothesis is supported by studies that have reported increased susceptibility to skin HSV infection in patients with atopic dermatitis, another inflammatory condition characterized by immune dysregulation and often coexisting with atopic conjunctivitis [15,31]. Furthermore, several immune pathways affect both the HSV keratitis reactivation rate and atopic conjunctival disease severity, possibly explaining the correlation between the conditions. A higher corneal concentration of dendritic cells, which are one of the hallmarks of atopic conjunctival disease as well [32], has been shown to correlate with higher rates of HSV keratitis reactivation and corneal opacification [21]. Autoreactive T lymphocytes, another hallmark of conjunctival atopic disease [33], have also been demonstrated in eyes with HSV keratitis reactivation [21]. The use of topical immunomodulatory treatment for atopic conjunctival disease (steroids, cyclosporin, tacrolimus) is another significant factor contributing to immune dysregulation of the ocular surface in these patients. Accordingly, this study demonstrated a significantly higher risk of HSV keratitis recurrence in the severe atopic conjunctivitis group, which may be attributable to the higher rate of routine immunomodulatory treatment utilization in these patients [34]. Finally, eye rubbing, which is often reported in patients with atopic conjunctival disease, has been shown to induce ocular inflammation as well [35]. A marked inflammatory palpebral conjunctival response has been observed in animal studies of eye rubbing [36,37]. Furthermore, eye rubbing, whether manual or caused by the typical papilla present on the inner eyelid’s surface in atopic conjunctival disease, elevates the corneal temperature. This in turn further increases the local inflammatory process and hyperemia [35], creating a vicious cycle further amplifying ocular inflammation.

Patients with atopic conjunctival disease often experience ocular surface damage and compromise, which could also render the cornea more susceptible to viral infections, including HSV [21]. The damage to the ocular surface may occur in this context due to either inflammatory and enzymatic damage to the corneal epithelium [38], inadvertent chafing of the cornea by papillae on the tarsal surface of the eyelid [38], or by manual eye rubbing which is prevalent in this population [38,39]. Various causes of trauma to the ocular surface have been described as a risk factor for HSV keratitis recurrence [10,11,12,13,21,40], including miscellaneous and medically induced conditions such as refractive surgery.

Thus, the combination of enzymatic and mechanical damage caused to the ocular surface in atopic conjunctival disease creates a favorable environment for HSV replication and persistence.

Interestingly, our results did not show a significant association between recurrent herpetic keratitis and other potential risk factors such as autoimmune disease, diabetes mellitus, human immunodeficiency virus (HIV) infection, and atopic dermatitis. HIV and autoimmune conditions (especially due to the immunomodulatory treatment they require) have been implicated as risk factors for dermatologic HSV infection and recurrence [41]. The lack of such an association of these conditions with ocular HSV recurrence suggests that atopic conjunctival disease may play a unique role in the development of recurrent HSV keratitis, possibly due to the direct involvement of the ocular surface in the inflammatory process and topical immunomodulating drugs which are often used in the management of atopic conjunctival disease. However, further studies are needed to elucidate the specific relationship between these conditions and to identify other factors that may modulate the risk of HSV keratitis in patients with atopic conjunctival disease.

The revelation of a significant association between atopic conjunctivitis and recurrent herpes keratitis in our study holds profound implications for both clinical practice and the broader understanding of ocular diseases. Recognizing atopic conjunctivitis as a potential predisposing factor for recurrent herpes keratitis informs clinicians about the need for heightened vigilance and tailored preventive strategies in this subset of patients. HSV keratitis is typically treated with topical (trifluridine, acyclovir, ganciclovir) or oral (acyclovir or valcyclovir) antiviral agents combined with topical steroids at varying doses according to stromal or uveitic involvement [2,7,42]. In cases with a suspected history of HSV keratitis and concomitant atopic conjunctival disease, the clinician must keep in mind the need for rapid and effective disease control as well as the need for oral preventive antiviral treatment when immunomodulating topical treatment is prescribed. The current indications for preventive antiviral treatment for HSV keratitis include two HSV keratitis exacerbations over a one-year period and corneal scar/vascularization formation approaching the visual axis postoperatively or during immunosuppressive treatment (topical or systemic) in any patient with a history of HSV keratitis [26]. Based on the findings of the current study, where atopic conjunctival disease increased the risk of recurrent HSV keratitis by 4.30 to 50.41-fold, we believe that prophylactic antiviral treatment ought to be considered for these patients as well. Long-term (one year), low-dose antiviral agents (acyclovir 400 mg twice daily or valacyclovir 500 mg once daily) have been proven to reduce the incidence of recurrent HSV keratitis by 50% during treatment [26]. It is also reasonable to extrapolate from existing genital herpes recurrence studies that famciclovir 250 mg twice daily may also be used for prophylaxis [26]. Such prophylaxis may aid in preventing the visual impairment as well as functional disturbance arising from recurrent HSV keratitis episodes in this predisposed population. Finally, patients with a history of HSV keratitis and concurrent atopic conjunctival disease should be educated regarding their relative risk of recurrence, acquainted with the signs and symptoms of recurrence, and informed that they should consult an ophthalmologist promptly if they experience warning signs or symptoms, since early detection and appropriate treatment are critical to minimize permanent visual loss [26].

This study has several limitations. The ophthalmological evaluations leading to the diagnosis of recurrent HSV keratitis were not available to the researchers; thus, the methods by which the diagnosis was established for each patient were not standardized. Due to the study design and the FFS coding system, data regarding disease severity and bilaterality were limited. Since no FFS code was assigned for a single HSV keratitis episode, data regarding these patients were not available either. This missing information could further shed light on the strength of association between HSV keratitis incidence and atopic conjunctival disease. In addition, it was not possible to confirm a direct causative relationship between atopic conjunctivitis and the HSV recurrence rate, since the FFS code was assigned at enrollment, with no information regarding which disease was diagnosed first in a given patient. Finally, the proposed mechanism connecting the two entities requires further scientific validation. Further in vivo and prospective clinical studies are needed in this field. It is also crucial to assess the cost-effectiveness of the proposed prophylactic treatment for HSV keratitis in patients with atopic conjunctival disease, as well as its safety, since the aforementioned clinical trials examining prophylaxis of HSV keratitis dealt with an adult population rather than a pediatric–young adolescent population where atopic conjunctival disease is very prevalent.

## 5. Conclusions

In summary, this study sheds light on the significant association between atopic conjunctival disease and recurrent herpetic keratitis in a large population-based cohort of immunocompetent adolescents and young adults. Patients with atopic conjunctival disease were 10.60-fold more likely to be diagnosed with recurrent herpetic keratitis compared to the general population. This risk increased even further, reaching 50.41-fold in patients with severe atopic conjunctival disease. It appears that the intricate relationship between atopic conjunctival disease and HSV keratitis is multifaceted, involving recurrent trauma to the corneal surface, chronic inflammation, immune dysregulation, and the use of immunomodulatory treatments.

The clinical implications of these findings are profound. Recognizing atopic conjunctival disease as a potential predisposing factor for recurrent HSV keratitis underscores the importance of tailored preventive strategies and heightened vigilance in this subset of patients. The study advocates for considering prophylactic antiviral treatment in cases where atopic conjunctival disease increases the risk of recurrent HSV keratitis, potentially preventing visual impairment and functional disturbance. Prospective clinical studies and further scientific validation are crucial to substantiate the proposed mechanisms and assess the cost-effectiveness and safety of prophylactic treatment.

## Figures and Tables

**Table 1:** Demographic characteristics of cases with and without recurrent herpetic keratitis. cm, centimeters; kg, kilograms; HIV, human immunodeficiency virus. * Vasculitis, rheumatic disease, inflammatory bowel disease, unspecified autoimmune disease.

	Recurrent HSV Keratitis (N = 160)	No Recurrent HSV Keratitis (N = 940,732)	*p* Value
Age (years)	17.80 ± 1.44	17.57 ± 1.50	0.071
Male gender	113 (70.63%)	557,486 (59.27%)	0.003
Height (cm)	169.45 ± 8.21	169.01 ± 8.94	0.568
Weight (kg)	64.89 ± 13.39	65.06 ± 14.56	0.891
Years of education	12.34 ± 2.82	12.32 ± 5.00	0.961
Cognitive function score	49.40 ± 20.17	52.22 ± 19.48	0.077
Autoimmune disease *	1 (0.63%)	2748 (0.29%)	0.374
Diabetes Mellitus	1 (0.63%)	4563 (0.49%)	1.000
HIV	0	39 (0.004%)	-
Atopic dermatitis	1 (0.63%)	13,585 (1.44%)	0.734

## Data Availability

Data is unavailable due to privacy or ethical restrictions.
